# Involvement of and Interaction between *WNT10A* and *EDA* Mutations in Tooth Agenesis Cases in the Chinese Population

**DOI:** 10.1371/journal.pone.0080393

**Published:** 2013-11-27

**Authors:** Huiying He, Dong Han, Hailan Feng, Hong Qu, Shujuan Song, Baojing Bai, Zhenting Zhang

**Affiliations:** 1 Department of Prosthodontics, School and Hospital of Stomatology, Peking University, Beijing, China; 2 College of Life Sciences, Peking University, Beijing, China; 3 Department of Genetics, Peking University Health Science Center, Beijing, China; 4 Department of Prosthodontics, School of Stomatology, Capital Medical University, Beijing, China; Instituto de Ciencia de Materiales de Madrid - Instituto de Biomedicina de Valencia, Spain

## Abstract

**Background:**

Dental agenesis is the most common, often heritable, developmental anomaly in humans. Although *WNT10A* gene mutations are known to cause rare syndromes associated with tooth agenesis, including onycho-odontodermal dysplasia (OODD), Schöpf-Schulz-Passarge syndrome (SSPS), hypohidrotic ectodermal dysplasia (HED), and more than half of the cases of isolated oligodontia recently, the genotype-phenotype correlations and the mode of inheritance of *WNT10A* mutations remain unclear. The phenotypic expression with *WNT10A* mutations shows a high degree of variability, suggesting that other genes might function with *WNT10A* in regulating ectodermal organ development. Moreover, the involvement of mutations in other genes, such as *EDA*, which is also associated with HED and isolated tooth agenesis, is not clear. Therefore, we hypothesized that *EDA* mutations interact with *WNT10A* mutations to play a role in tooth agenesis. Additionally, *EDA*, *EDAR*, and *EDARADD* encode signaling molecules in the Eda/Edar/NF-κB signaling pathways, we also checked *EDAR* and *EDARADD* in this study.

**Methods:**

*WNT10A*, *EDA*, *EDAR* and *EDARADD* were sequenced in 88 patients with isolated oligodontia and 26 patients with syndromic tooth agenesis. The structure of two mutated WNT10A and two mutated EDA proteins was analyzed.

**Results:**

Digenic mutations of both *WNT10A* and *EDA* were identified in 2 of 88 (2.27%) isolated oligodontia cases and 4 of 26 (15.38%) syndromic tooth agenesis cases. No mutation in *EDAR* or *EDARADD* gene was found.

**Conclusions:**

*WNT10A* and *EDA* digenic mutations could result in oligodontia and syndromic tooth agenesis in the Chinese population. Moreover, our results will greatly expand the genotypic spectrum of tooth agenesis.

## Introduction

Permanent tooth agenesis is the most common developmental dental anomaly in humans, with an incidence rate of 2.2–10.1% in the general population (excluding anomalies of the third molars) [1]. Permanent tooth agenesis can occur as an isolated anomaly (non-syndromic) or as a part of multiple congenital anomalies (syndromic) [2]–[4], such as anhidrotic or hypohidrotic ectodermal dysplasia (HED), onycho-odontodermal dysplasia (OODD) and Schöpf-Schulz-Passarge syndrome (SSPS). Selective tooth agenesis is divided into 2 types: hypodontia, the agenesis of fewer than six teeth, and oligodontia, the agenesis of six or more permanent teeth.

Mutations of the *WNT10A* gene are responsible for OODD, HED and SSPS [5]–[7]. OODD, a rare form of ectodermal dysplasia, is characterized mainly by abnormal teeth, nail dystrophy, palmoplantar keratoderma, hypotrichosis and various other associated ectodermal abnormalities, including neoplasms [5]. HED is characterized by a triad of signs comprising sparse hair (hypotrichosis), abnormal or missing teeth (anodontia or hypodontia), and inability to sweat (anhidrosis or hypohidrosis) [6]. SSPS is distinguished by the presence of multiple eyelid cysts, histologically corresponding to apocrine hidrocystomas [7].

WNT10A, a 46.4-kDa protein with 10 putative α-helices and seven putative β-strands, belongs to Wnt proteins, a large family of secreted signaling proteins. Wnt10a is expressed in embryonic limb [8], skin [9], teeth [10], [11], and hair follicles [12], [13] during embryonic development; it plays an important role in odontoblast differentiation and tooth morphogenesis [11]. Recently, *WNT10A* mutations were identified in more than half of isolated hypodontia cases [14]. However, the phenotypic expression of *WNT10A* mutations shows a high degree of variability, ranging from only isolated hypodontia to various symptoms of ectodermal dysplasia. Moreover, the genotype-phenotype correlations and the mode of inheritance of *WNT10A* mutations remain unclear [6], [14], [15]. Therefore, other genetic factors or unidentified mutations may also influence phenotypic expression in patients with *WNT10A* mutations [6], [14].

*EDA* is an important gene associated with tooth agenesis, it is located on chromosome Xq12–q13.1 and encodes for ectodysplasin-A (EDA) (MIM 300451), a member of the tumor necrosis factor (TNF) family [16]. EDA is a type II transmembrane protein with a C-terminal TNF homology domain and a signaling molecule [17]. *EDAR* and *EDARADD* encode the protein EDAR and EDARADD, belonging to the Eda/Edar/NF-κB signaling pathway. The binding of Eda-Edar (complex formed by EDA and its receptor) to the downstream adaptor Edaradd leads to activation of the transcription factor NF-κB and is essential for the development of hair follicles, teeth, exocrine glands and other ectodermal derivatives [17]–[19]. *EDA* gene mutations have been detected in 63–95% of X-Linked HED (XLHED) patients [16], [20]–[23]. Moreover, *EDA* mutations are also associated with cases of isolated tooth agenesis [24], [25]. We therefore hypothesized that *EDA* mutations may interact with *WNT10A* mutations and play a role in the development of tooth agenesis.

In this study, we investigated the contribution of *WNT10A*, *EDA, EDAR* and *EDARADD* mutations in patients with isolated or syndromic tooth agenesis. Our results suggest that *WNT10A* and *EDA* digenic mutations could result in tooth agenesis. This is the first time such mutations have been reported in patients with tooth agenesis.

## Materials and Methods

### Participants

Written informed consent for DNA analysis and reproduction of the photographs was obtained from all the participants and the parents on the behalf of the minors or children participants. This study was conducted with the approval of the Ethics Committee of Peking University Health Science Center.

The study participants were 88 non-consanguineous patients with isolated tooth agenesis, 26 non-consanguineous patients with syndromic tooth agenesis (24 with HED and 2 with OODD), and 451 non-consanguineous normal controls, who were referred to the Department of Prosthodontics, Peking University School and Hospital of Stomatology, or the Department of Prothodontics, Beijing Stomatological Hospital. Oral examinations for all participants were performed by a prosthodontist, who determined the status of the dentition. A panoramic radiograph was taken to confirm the diagnosis of tooth agenesis for these participants. The shape and size of the residual teeth were also observed.

The patients with isolated dental agenesis reported normal sweating and lachrymal secretions. They had no complaints about dry mouth, intolerance to heat, or susceptibility to respiratory tract infections. The patients had hair on their body and scalp, and their facial features, skin, and nails were normal on observation.

Patients with HED presented abnormalities of at least two of the three following ectodermal structures: teeth, hair and sweat glands. The OODD patients had abnormal teeth, nail dystrophy, palmoplantar keratoderma and other associated ectodermal abnormalities.

### DNA extraction

Genomic DNA of the participants and their relatives was extracted from peripheral blood lymphocytes using a QIAamp DNA Blood Midi Kit (Qiagen). DNA samples of the normal participants recruited from the general population were extracted from buccal epithelial cells using the Chelex-100 (Sigma) method.

### Detection of mutations

Screening of the *WNT10A*, *EDA*, *EDAR*, and *EDARADD* genes was performed by direct sequencing of five PCR fragments for *WNT10A*, eight PCR fragments for *EDA*, ten PCR fragments for *EDAR*, and eight PCR fragments for *EDARADD*, which cover the entire cDNA including exons and intron-exon junctions of more than 100 base pairs. We compared all primer sequences to the whole-genome assembly in the ENSEMBL database to verify their uniqueness against gene families. Primer sequences are available upon request.

### Protein structure analysis

We performed protein structure analysis on the two *WNT10A* mutations (p.R171C and p.G213S) and two novel *EDA* mutations (p.G257R and p.I312M) that were identified in this study.

For *WNT10A*, the conservation of residues in sequences was determined to predict the influence of the two mutations. The sequences of orthologs of human WNT10A protein were retrieved from the KEGG database [26]; these sequences were used to perform sequence alignment with ClustalX2.0.12 [27]. PsiPred 3.0 [28] was used to predict the 2D structure of human WNT10A protein.

We used the crystal structure of EDA-A1 (PDB id 1RJ7 [29]) as a scaffold to predict the influence of the mutations. The structures were analyzed using the Insight II (2000) software package (Accelrys Inc., San Diego, CA, USA). Images were produced by PyMOL (DeLano WL., 2002.The PyMOL Molecular Graphics System, DeLano Scientific, Palo Alto, CA, USA. http://www.pymol.org) from PDB file 1RJ7.

## Results

**Figure 1:**
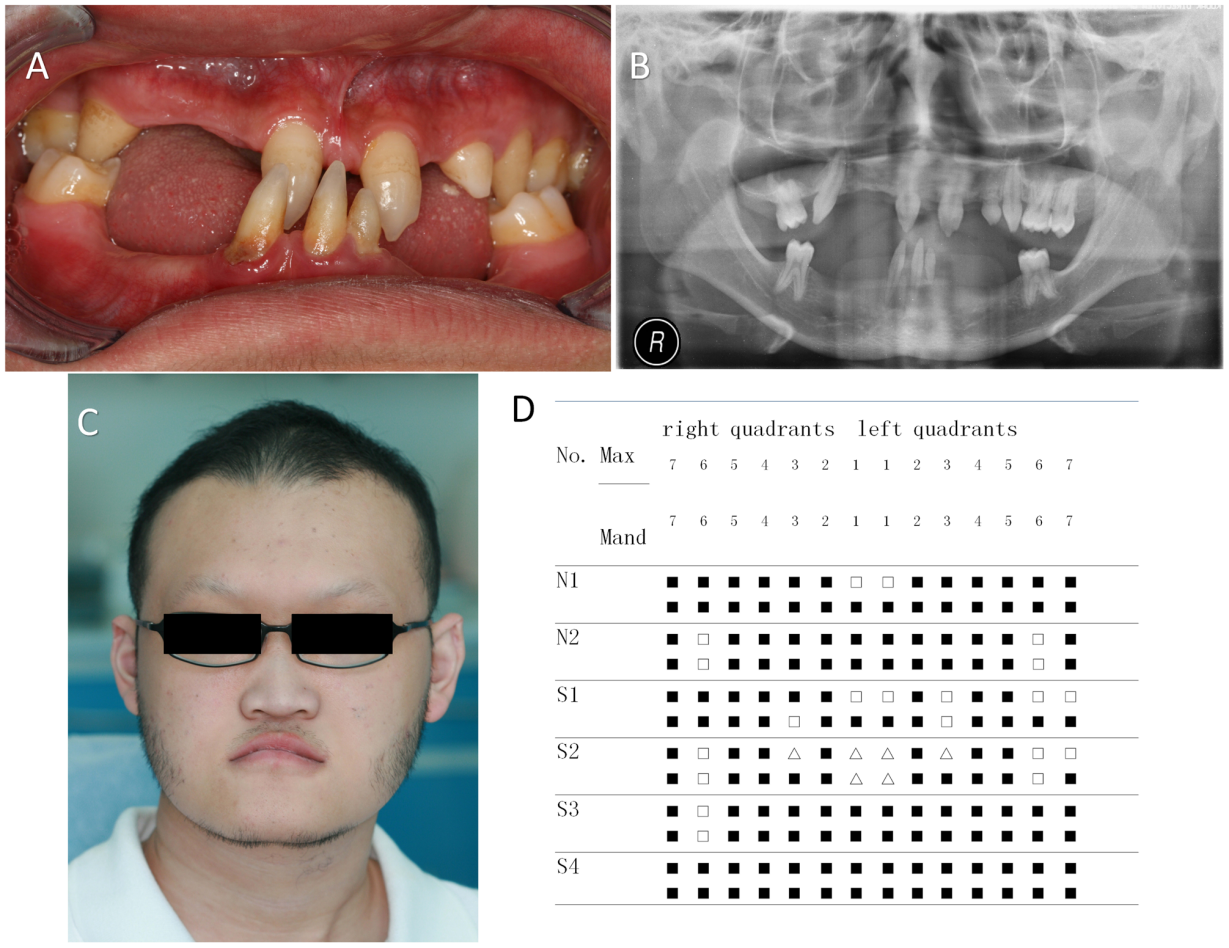
Clinical characteristics of patients with tooth agenesis with digenic mutations in both *WNT10A* and *EDA* (**A**) Clinical phenotype of patient S2 showing congenital tooth agenesis. (**B**) Panoramic radiograph of the dentition of patient S2. (**C**) Facial profile of patient S2. (**D**) Schematic presentation of congenitally missing teeth of the patients with digenic mutations, two with non-syndromic oligodontia and four with syndromic tooth agenesis. The missing tooth is represented by a filled square; △, tapered tooth; Max, maxillary; Mand, mandibular.

Digenic mutations of both *EDA* and *WNT10A* were identified in 2 of 88 (2.27%) isolated oligodontia cases and 4 of 26 (15.38%) syndromic tooth agenesis cases. The patients in all four syndromic cases had HED (Fig. 1). No mutation in *EDAR* or *EDARADD* gene was found, however, some single nucleotide polymorphism (SNP) loci were detected (Table S1).

### Clinical details and DNA sequence analysis of six nuclear families with digenic mutations

**Figure 2:**
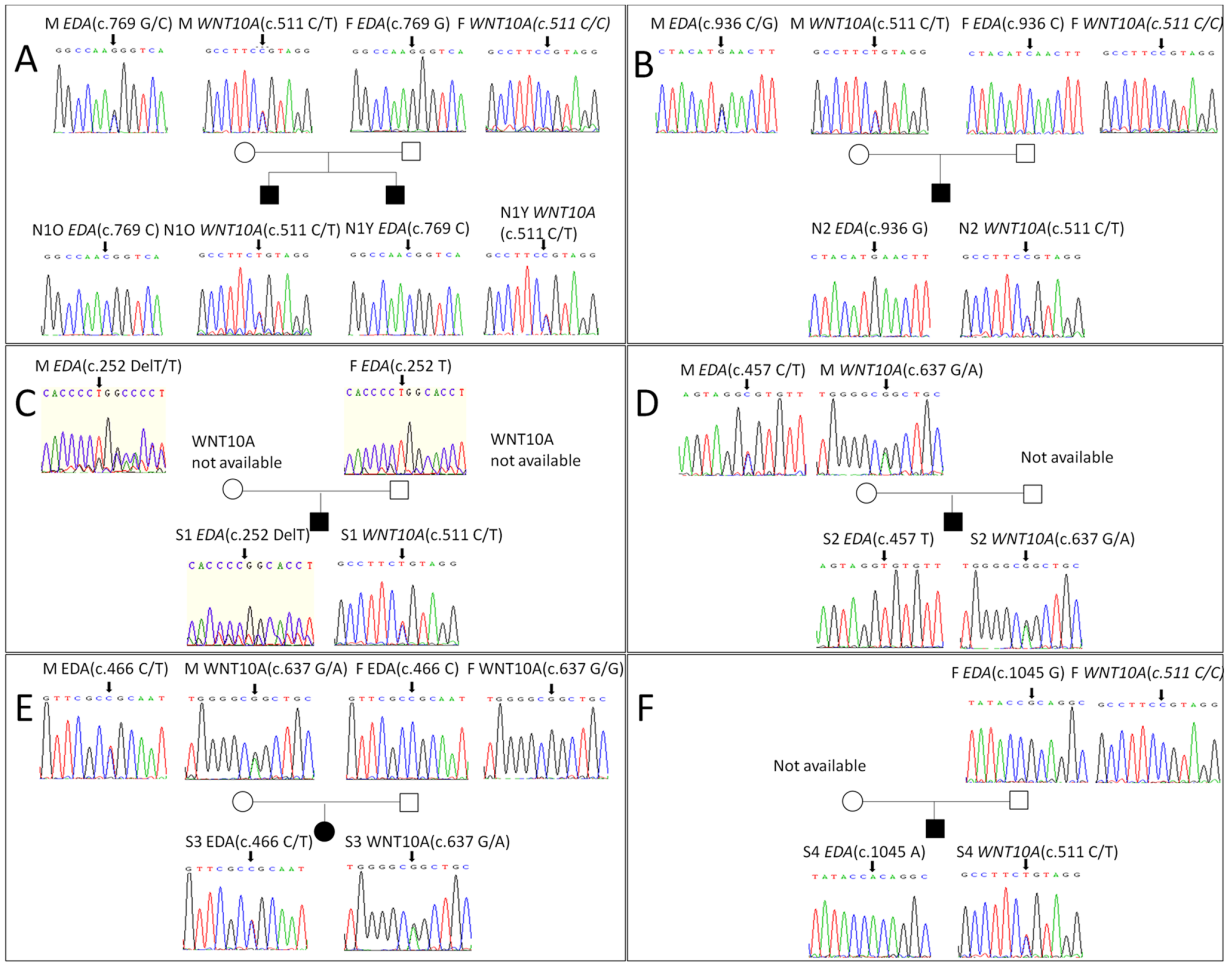
Sequence analyses of *EDA* and *WNT10A* genes. (**A**) The *EDA* mutation c.769G>C and *WNT10A* mutation c.511C>T were found in patient N1, who inherited the mutant allele from his mother. (**B**) The *EDA* mutation c.936C>G and *WNT10A* mutation c.511C>T were found in patient N2, who also inherited the mutant allele from his mother. (**C**) The *EDA* mutation c.252DelT and *WNT10A* mutation c.511C>T were found in patient S1, who inherited the mutant *EDA* allele from his mother; *WNT10A* mutations in the parents could not be analyzed. (**D**) The *EDA* mutation c.457C>T and *WNT10A* mutation c.637G>A were found in patient S2, who also inherited the mutant allele from his mother; however, his father's DNA sample could not be obtained for analysis. (**E**) The *EDA* mutation c.466C>T and *WNT10A* mutation c.637G>A were found in patient S3, who inherited the mutant allele from his mother. (**F**) The mutations c.1045G>A in *EDA* and c.511C>T in *WNT10A* were found in patient S4, but his mother's DNA sample could not be obtained. All mutated nucleotides are identified by arrows. M, mother; F, father; O, older brother; Y, younger brother.

“N1” in the non-syndromic group (Table 1) represents a pair of male monozygotic twins (4 years old) who were considered as one participant. The number of missing teeth in both boys was 26. The nucleotide sequence showed a G to C transition at nucleotide 769 (c.769G>C) of the coding sequence in exon 7 of *EDA*, which results in the substitution of Gly at residue 257 to Arg. Additionally, the nucleotide sequence showed a monoallelic C to T transition at nucleotide 511 (c.511C>T) of the coding sequence in exon 3 of *WNT10A*, which results in the substitution of Arg at residue 171 to Cys. DNA sequencing of the parents' genome revealed that both mutant alleles were from their mother (Fig. 2A), who carried a heterozygous *EDA* mutation (c.769G>C) and a heterozygous *WNT10A* c.511C>T mutation, and showed absence of only the left upper lateral incisor without other clinical abnormalities. No mutations in these genes were found in the father.

**Table 1:** Clinical features and mutations in the six patients.

Group	Proband	Gender	Age	EDA	WNT10A	Number of missing teeth	Hair	Dry skin	Hypohidrosis/anhidrosis	Plantar hyperkeratosis	Nails	Other signs
Non-syndromic tooth agenesis	N1	male	4	p.G257R (c.769G>C) *	p.R171C(c.511C>T)	26	curl	-	-	-	rough	-
	N2	male	6	p.I312M (c.936C>G) *	p.R171C(c.511C>T)	24	-	-	-	-	-	-
Syndromic tooth agenesis	S1	male	14	p.P84PfsX6(c.252DelT)	p.R171C(c.511C>T)	21	+	-	+	-	-	-
	S2	male	17	P.R153C(c.457C>T)	p.G213S(c.637G>A)	17	+-,curl	-	+	-	-	-
	S3	female	14	p.R156C(c.466C>T)	p.G213S(c.637G>A)	26	+	+	+	-	-	Eczema
	S4	male	8	p.A349T(c.1045G>A)	p.R171C(c.511C>T)	28	+	+	+	-	-	Dry eyes

M, male; F, female; +, present; _, absent; +_, very mild; *, Novel mutations identified in the present study.

“N2” is a 6-year-old boy who was normal except for the absence of 24 teeth (Table 1). The p.Ile312Met (c.936C>G) mutation in *EDA* and heterozygous p.Arg171Cys (c.511C>T) mutation in *WNT10A* were detected. The coding sequence in exon 9 of *EDA* showed a C to G transition, which results in the substitution of Ile at residue 312 to Met; also, the coding sequence in exon 3 of *WNT10A* showed a C to T transition at nucleotide 511, which results in the substitution of Arg at residue 171 to Cys. Analyses of his parents' genome revealed that the mutant alleles were from his mother, who carried digenic heterozygous *EDA* and *WNT10A* mutations at the same locus as that of N2 (Fig. 2B). Clinical examination showed that maxillary lateral incisors on both sides and the left mandibular second molar were missing in the mother, but there were no anomalies in other organs. The father did not have any mutations for these genes.

“S1” is a 14-year-old boy who had 21 permanent teeth missing (Table 1). The nucleotide sequence showed a T deletion at nucleotide 252 (c.252DelT) of the coding sequence in exon 1 of *EDA*; this leads to a frame shift from residue 84 and a premature termination at residue 90. Additionally, a monoallelic C to T transition at nucleotide 511 (c.511C>T) of the coding sequence in exon 3 of *WNT10A* was detected, this leads to the substitution of Arg at residue 171 to Cys. Analyses of his parents' genome showed that the mutant *EDA* allele was from his mother (Fig. 2C), however, we were unable to screen for *WNT10A* mutations because of insufficient DNA.

“S2” is a 17-year-old boy who had curly hair, 17 missing permanent teeth and hypohidrosis, his skin and nails were normal (Fig. 1 and Table 1). The p.Arg153Cys (c.457C>T) mutation was found in exon 3 of *EDA*, it results in the substitution of Arg at residue 153 to Cys. Moreover, a heterozygous p.Gly213Ser (c.637G>A) mutation was detected in exon 3 of *WNT10A*, this leads to the substitution of Gly at residue 213 to Ser. Sequence analyses revealed that both mutant alleles were from his mother (Fig. 2D), who had a very mild phenotype of isolated tooth agenesis. His father did not have mutations in either of these genes.

“S3” is a 14-year-old girl who had the typical clinical characteristics of HED: sparse hair, 26 missing permanent teeth, hypohidrosis, dry skin, and eczema on her body, but no plantar hyperkeratosis or nail abnormalities (Table 1). The heterozygous p.Arg156Cys (c.466C>T) mutation was found in exon 3 of *EDA*, it results in the substitution of Arg at residue 156 to Cys. Additionally, the monoallelic p.Gly213Ser (c.637G>A) mutation was also detected in exon 3 of *WNT10A*, it results in the substitution of Gly at residue 213 to Ser. Sequence analyses of her parents' genome revealed that the mutant alleles were from her mother (Fig. 2E), who only had microdontia of the upper lateral incisors. Her father did not carry mutations for either of these genes.

“S4” is an 8-year-old boy who also had the typical characteristics and facial features of HED and was missing 28 permanent teeth, but he did not have plantar hyperkeratosis or nail abnormalities (Table 1). The p.Ala349Thr (c.1045G>A) mutation in exon 9 of *EDA* and heterozygous p.Arg171Cys (c.511C>T) mutation in exon 3 of *WNT10A* were detected. These mutations were not found in his father's genome, but because his mother's DNA sample was unavailable, the origin of the mutant alleles was not clear (Fig. 2F).

All novel mutations that were identified in this study were not found in the normal controls.

### Protein structure analysis

**Figure 3:**
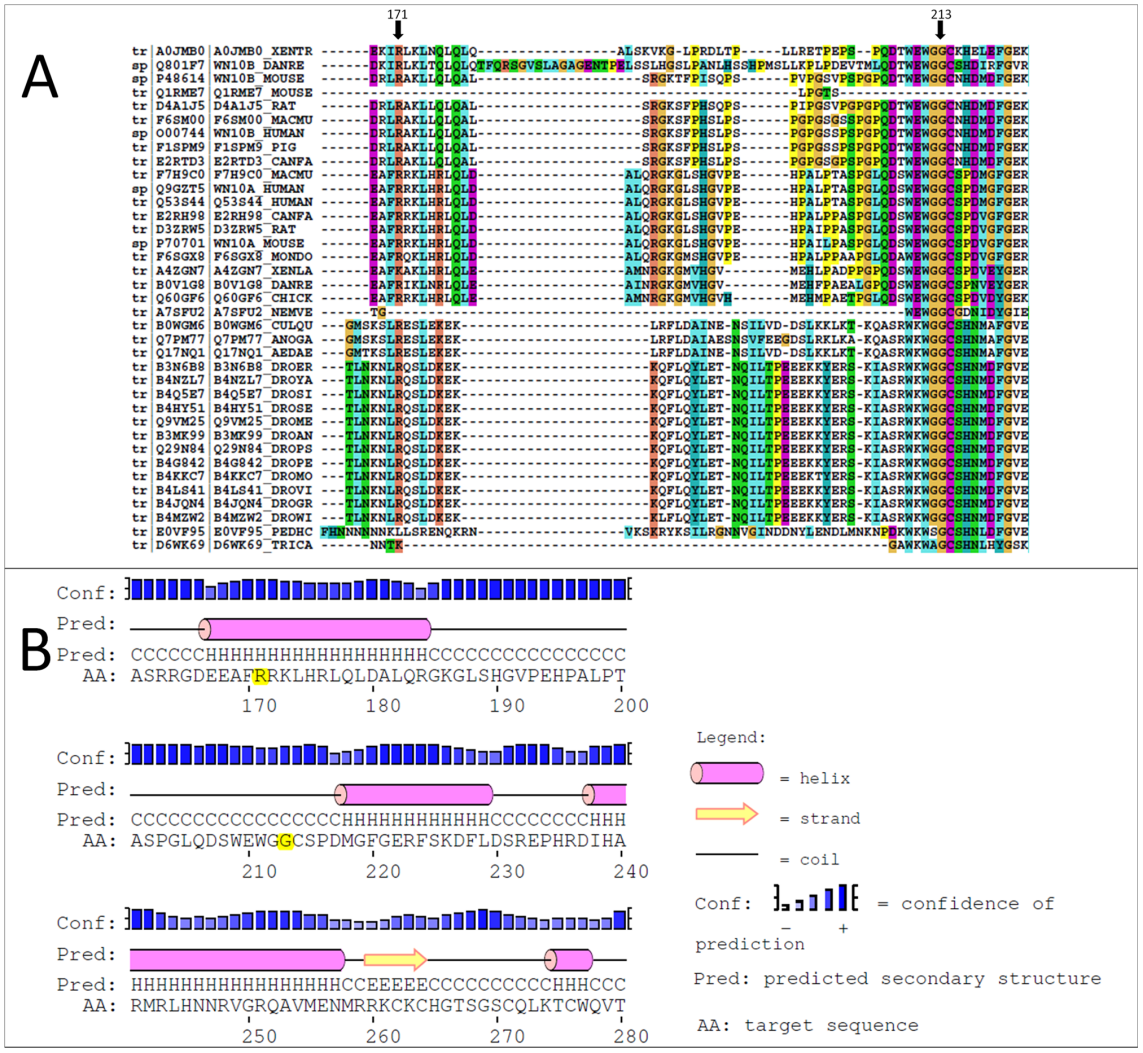
Sequences of orthologs and predicted 2D structure of human WNT10A protein. (**A**) The alignment of orthologs of the human WNT10A protein. The R171 and G213 residues are represented by arrowheads. (**B**) The predicted 2D structure of human WNT10A protein. The R171 and G213 residues are in yellow.

The results of protein structure analyses of WNT10A are shown in Figure 3. R171 and G213 are conserved residues through these organisms and located on conserved 2D fragments. Mutations of the residues could affect the function of the human WNT10A protein. In the case of R171C mutations, the substitution of Cys, a hydroxylic amino acid with a side chain shorter than Arg, might eliminate the electrostatic interaction of R171 with adjacent residues. The mutation G213S is expected to abolish the hydrophobic interaction of G213 with adjacent residues.

**Figure 4:**
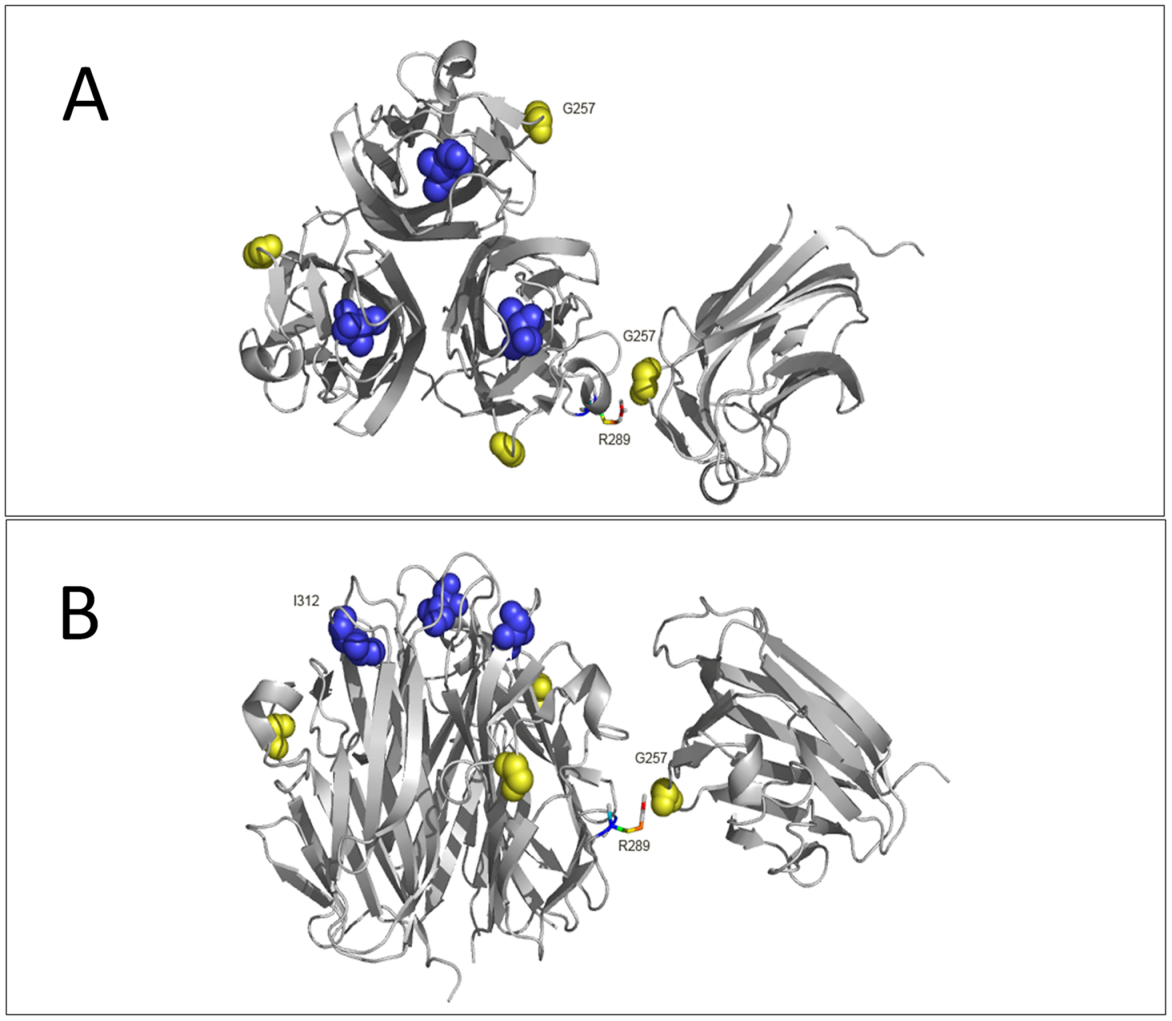
Structure analysis of mutant residues in the three-dimensional EDA trimer. The EDA trimer is shown as a ribbon with relevant side chains rendered in spheres. The G257 and I312 residues are in yellow and blue, respectively. The side chain of the R289 residue is represented by a colored stick. (**A**) The planform of the EDA trimer. (**B**) The side view of the EDA trimer.

The 3D structure of EDA is shown in Figure 4. The G257 residue is located at the interface of two trimers. When G257R mutation happened, the side chain volume significantly enlarged, making it possible to form interaction with the R289 in adjacent trimer and abolish the stabilization of EDA. I312 is located at the outer surface of the three monomers. An I312M mutation could affect the interactions of EDA with its receptors.

## Discussion

This is the first study to show that simultaneous *WNT10A* and *EDA* mutations could lead to tooth agenesis in the Chinese population. We found that six participants harbored digenic mutations in both *WNT10A* and *EDA*: two of them had isolated oligodontia and the others had syndromic tooth agenesis. Two *WNT10A* and six *EDA* mutations were identified in these six patients. Two of the *EDA* mutations, p.G257R and p.I312M, which were identified in isolated oligodontia patients, are novel mutations. Based on the results of three-dimensional EDA structure analysis, we think that the two novel *EDA* mutations cause functional abnormalities in the EDA protein; therefore, we consider these two *EDA* mutations to be pathogenic. We used the sequence alignment of the orthologs to predict the 2D structure of human WNT10A protein; based on the results, we think that the two *WNT10A* mutations are highly conserved residues and that these mutations could affect the function of human WNT10A protein. The other four *EDA* mutations identified in HED patients and the two *WNT10A* mutations have been reported in tooth agenesis patients previously [20], [30], [31].

Using phenotype-genotype analysis, we found that although patient S3 and her mother had the same mutation (heterozygous *EDA* p.Arg156Cys mutation and heterozygous *WNT10A* p.Gly213Ser mutation), patient S3 showed typical phenotypic expression of HED while her mother only had microdontia. Because the *EDA* mutation is the most frequent pathogenic cause of XLHED and the *EDA* gene is located on chromosome X, most female carriers of *EDA* mutations present with a normal or very mild phenotype [32], [33]. Therefore, one possible explanation for the differential expression of the phenotypes in patient S3 and her mother could be random X chromosome inactivation.

In this study, patients N1, N2 and S2 had hemizygous *EDA* mutations and heterozygous *WNT10A* mutations; their mutations were from their mothers, who harbored composite heterozygous mutations of both *EDA* and *WNT10A*. Therefore, the heterozygous *WNT10A* mutation was identical between the patient and mother, but the *EDA* mutation was not. If we consider this and the observation of the more severe phenotype in the patients together, it seems that the differences in the *EDA* mutation may have been responsible for the phenotype differences. This also means that heterozygous *WNT10A* mutations may result in a very mild phenotype. Moreover, the phenotype of these patients was not more distinctly severe than those of patient with single *EDA* mutations reported previously [6], [32], which further supports our speculation. However, further studies are definitely required to confirm these speculations, possibly in a bigger patient group.

Previous studies have indicated that digenic mutations in one patient could cause two different diseases. For example, Wang et al. reported the case of a 5-year-old boy who had dentin defects caused by a *COL1A2* mutation and hypodontia caused by a *PAX9* mutation [34]. On the other hand, two different gene mutations could influence each other and result in one disease: for example, *Desmoglein-2* and *Desmocollin-2* mutations have been reported to cause arrhythmogenic right ventricular dysplasia/cardiomyopathy [35]; a *OGG1* mutation in combination with a *MUTYH* mutation reportedly led to hereditary colorectal cancer [36]; and *NEK1* and *DYNC2H1* mutations resulted in short rib polydactyly, Majewski type [37]. In this study, digenic mutations of both *WNT10A* and *EDA* caused isolated and syndromic tooth agenesis, and the protein structure analyses indicated that all the *WNT10A* and *EDA* mutations may affect protein function. Therefore, we think that interaction between two mutated proteins may cause tooth developmental anomalies.

Wnt10a functions through the canonical Wnt/β-catenin pathway, which plays crucial roles at multiple stages of tooth development. Interruption of the Wnt/β-catenin pathway results in severe tooth agenesis [38]. Eda functions via the Eda/Edar/NF-κB signaling pathways. Reciprocal interaction between the Wnt/β-catenin and Eda/Edar/NF-κB signaling pathways continues throughout the process of ectodermal organ development. In developing skin appendages, Dkk4, which acts as an inhibitor of Wnt signaling, is the direct transcriptional target of Eda/Edar signaling during placode formation [39]. Zhang et al. recently showed a sequential interdependency between the Wnt and Eda pathways in developing hair follicles: Wnt/β-catenin signaling is essential for NF-κB activation, whereas Edar/NF-κB is thereafter required to strengthen and maintain Wnt/β-catenin activity [13]. Moreover, during tooth morphogenesis, Wnt10a and Edar were expressed at the same locations before the cap stage, that is, in the dental epithelium at initiation and bud stages and in the enamel knot during the cap stage [11]. Wnt signals regulate ectodysplasin expression in the oral ectoderm, and the expression of Edar in the epithelial signaling centers is responsive to Wnt-induced ectodysplasin from the nearby ectoderm [40]. Therefore, mutations in WNT10A and EDA proteins might affect these pathways and cause abnormalities in tooth development. However, further studies are required to elucidate the interaction between Eda/Edar/NF-κB and Wnt/β-catenin signaling in normal and abnormal tooth development.

In conclusion, this is the first study to report two simultaneous gene mutations of *EDA* and *WNT10A* in HED and isolated tooth agenesis patients. Our results suggest that *WNT10A* and *EDA* digenic mutations could result in oligodontia and syndromic tooth agenesis in the Chinese population. The results further confirm that other genetic factors influence the phenotypic expression in severe tooth agenesis patients with heterozygous *WNT10A* mutations. We believe that this study will greatly expand the genotypic spectrum of tooth agenesis.

## Supporting Information

Table S1 **Single nucleotide polymorphisms in** ***EDAR*** **and** ***EDARADD*** **of 114 patients.**
(PPT)

## References

[1] Polder BJ, Van't Hof MA, Van der Linden FPGM, Kuijpers-Jagtman AM (2004) A meta-analysis of the prevalence of dental agenesis of permanent teeth. Community Dentistry and Oral Epidemiology 32: 217–226. 15151692 10.1111/j.1600-0528.2004.00158.x

[2] Nieminen P (2009) Genetic basis of tooth agenesis. Journal of Experimental Zoology Part B: Molecular and Developmental Evolution 312B: 320–342. 10.1002/jez.b.21277 19219933

[3] Bailleul-Forestier I, Berdal A, Vinckier F, de Ravel T, Fryns JP, et al (2008) The genetic basis of inherited anomalies of the teeth. Part 2: syndromes with significant dental involvement. Eur J Med Genet 51: 383–408. 18599376 10.1016/j.ejmg.2008.05.003

[4] Bailleul-Forestier I, Molla M, Verloes A, Berdal A (2008) The genetic basis of inherited anomalies of the teeth. Part 1: clinical and molecular aspects of non-syndromic dental disorders. Eur J Med Genet 51: 273–291. 18499550 10.1016/j.ejmg.2008.02.009

[5] Adaimy L, Chouery E, Mégarbané H, Mroueh S, Delague V, et al (2007) Mutation in WNT10A Is Associated with an Autosomal Recessive Ectodermal Dysplasia: The Odonto-onycho-dermal Dysplasia. American journal of human genetics 81: 821–828. 17847007 10.1086/520064 PMC1973944

[6] Cluzeau C, Hadj-Rabia S, Jambou M, Mansour S, Guigue P, et al (2011) Only four genes (EDA1, EDAR, EDARADD, and WNT10A) account for 90% of hypohidrotic/anhidrotic ectodermal dysplasia cases. Human Mutation 32: 70–72. 20979233 10.1002/humu.21384

[7] Castori M, Ruggieri S, Giannetti L, Annessi G, Zambruno G (2008) Schopf-Schulz-Passarge syndrome: further delineation of the phenotype and genetic considerations. Acta Derm Venereol 88: 607–612. 19002348 10.2340/00015555-0547

[8] Narita T, Sasaoka S, Udagawa K, Ohyama T, Wada N, et al (2005) Wnt10a is involved in AER formation during chick limb development. Developmental Dynamics 233: 282–287. 15789446 10.1002/dvdy.20321

[9] Garriock RJ, Warkman AS, Meadows SM, D'Agostino S, Krieg PA (2007) Census of vertebrate Wnt genes: isolation and developmental expression of Xenopus Wnt2, Wnt3, Wnt9a, Wnt9b, Wnt10a, and Wnt16. Dev Dyn 236: 1249–1258. 17436276 10.1002/dvdy.21156

[10] Kratochwil K, Galceran J, Tontsch S, Roth W, Grosschedl R (2002) FGF4, a direct target of LEF1 and Wnt signaling, can rescue the arrest of tooth organogenesis in Lef1(-/-) mice. Genes Dev 16: 3173–3185. 12502739 10.1101/gad.1035602 PMC187508

[11] Yamashiro T, Zheng L, Shitaku Y, Saito M, Tsubakimoto T, et al (2007) Wnt10a regulates dentin sialophosphoprotein mRNA expression and possibly links odontoblast differentiation and tooth morphogenesis. Differentiation 75: 452–462. 17286598 10.1111/j.1432-0436.2006.00150.x

[12] Reddy S, Andl T, Bagasra A, Lu MM, Epstein DJ, et al (2001) Characterization of Wnt gene expression in developing and postnatal hair follicles and identification of Wnt5a as a target of Sonic hedgehog in hair follicle morphogenesis. Mech Dev 107: 69–82. 11520664 10.1016/s0925-4773(01)00452-x

[13] Zhang Y, Tomann P, Andl T, Gallant NM, Huelsken J, et al (2009) Reciprocal requirements for EDA/EDAR/NF-kappaB and Wnt/beta-catenin signaling pathways in hair follicle induction. Dev Cell 17: 49–61. 19619491 10.1016/j.devcel.2009.05.011 PMC2859042

[14] van den Boogaard M-J, Créton M, Bronkhorst Y, van der Hout A, Hennekam E, et al (2012) Mutations in WNT10A are present in more than half of isolated hypodontia cases. Journal of Medical Genetics 49: 327–331. 22581971 10.1136/jmedgenet-2012-100750

[15] Bohring A, Stamm T, Spaich C, Haase C, Spree K, et al (2009) WNT10A Mutations Are a Frequent Cause of a Broad Spectrum of Ectodermal Dysplasias with Sex-Biased Manifestation Pattern in Heterozygotes. American journal of human genetics 85: 97–105. 19559398 10.1016/j.ajhg.2009.06.001 PMC2706962

[16] Bayés M, Hartung AJ, Ezer S, Pispa J, Thesleff I, et al (1998) The Anhidrotic Ectodermal Dysplasia Gene (EDA) Undergoes Alternative Splicing and Encodes Ectodysplasin-A with Deletion Mutations in Collagenous Repeats. Human Molecular Genetics 7: 1661–1669. 9736768 10.1093/hmg/7.11.1661

[17] Mikkola ML, Thesleff I (2003) Ectodysplasin signaling in development. Cytokine Growth Factor Rev 14: 211–224. 12787560 10.1016/s1359-6101(03)00020-0

[18] Schmidt-Ullrich R, Aebischer T, Hulsken J, Birchmeier W, Klemm U, et al (2001) Requirement of NF-kappaB/Rel for the development of hair follicles and other epidermal appendices. Development 128: 3843–3853. 11585809 10.1242/dev.128.19.3843

[19] Orange JS, Levy O, Geha RS (2005) Human disease resulting from gene mutations that interfere with appropriate nuclear factor-kappaB activation. Immunol Rev 203: 21–37. 15661019 10.1111/j.0105-2896.2005.00221.x

[20] Monreal AW, Zonana J, Ferguson B (1998) Identification of a new splice form of the EDA1 gene permits detection of nearly all X-linked hypohidrotic ectodermal dysplasia mutations. Am J Hum Genet 63: 380–389. 9683615 10.1086/301984 PMC1377324

[21] Pääkkönen K, Cambiaghi S, Novelli G, Ouzts LV, Penttinen M, et al (2001) The mutation spectrum of the EDA gene in X-linked anhidrotic ectodermal dysplasia. Human Mutation 17: 349–349. 10.1002/humu.33 11295832

[22] Schneider P, Street SL, Gaide O, Hertig S, Tardivel A, et al (2001) Mutations Leading to X-linked Hypohidrotic Ectodermal Dysplasia Affect Three Major Functional Domains in the Tumor Necrosis Factor Family Member Ectodysplasin-A. Journal of Biological Chemistry 276: 18819–18827. 11279189 10.1074/jbc.M101280200

[23] Vincent MC, Biancalana V, Ginisty D, Mandel JL, Calvas P (2001) Mutational spectrum of the ED1 gene in X-linked hypohidrotic ectodermal dysplasia. Eur J Hum Genet 9: 355–363. 11378824 10.1038/sj.ejhg.5200635

[24] Han D, Gong Y, Wu H, Zhang X, Yan M, et al (2008) Novel EDA mutation resulting in X-linked non-syndromic hypodontia and the pattern of EDA-associated isolated tooth agenesis. Eur J Med Genet 51: 536–546. 18657636 10.1016/j.ejmg.2008.06.002

[25] Song S, Han D, Qu H, Gong Y, Wu H, et al (2009) EDA gene mutations underlie non-syndromic oligodontia. J Dent Res 88: 126–131. 19278982 10.1177/0022034508328627 PMC3317984

[26] Kanehisa M, Goto S, Furumichi M, Tanabe M, Hirakawa M (2010) KEGG for representation and analysis of molecular networks involving diseases and drugs. Nucleic Acids Res 38: D355–360. 19880382 10.1093/nar/gkp896 PMC2808910

[27] Larkin MA, Blackshields G, Brown NP, Chenna R, McGettigan PA, et al (2007) Clustal W and Clustal X version 2.0. Bioinformatics 23: 2947–2948. 17846036 10.1093/bioinformatics/btm404

[28] Buchan DW, Ward SM, Lobley AE, Nugent TC, Bryson K, et al (2010) Protein annotation and modelling servers at University College London. Nucleic Acids Res 38: W563–568. 20507913 10.1093/nar/gkq427 PMC2896093

[29] Hymowitz SG, Compaan DM, Yan M, Wallweber HJ, Dixit VM, et al (2003) The crystal structures of EDA-A1 and EDA-A2: splice variants with distinct receptor specificity. Structure 11: 1513–1520. 14656435 10.1016/j.str.2003.11.009

[30] Zhang J, Han D, Song S, Wang Y, Zhao H, et al (2011) Correlation between the phenotypes and genotypes of X-linked hypohidrotic ectodermal dysplasia and non-syndromic hypodontia caused by ectodysplasin-A mutations. Eur J Med Genet 54: e377–382. 21457804 10.1016/j.ejmg.2011.03.005

[31] Zhao R, Song S, He H, Feng H, Zhang J (2013) WNT10A Gene Mutation Cause Isolated Tooth Agenesis. IADR/AADR/CADR General Session and Exhibition: Poster Session-Abstract #2746. Available: http://iadr.confex.com/iadr/13iags/webprogram/Paper172937.html. Accessed 22 March 2013.

[32] Nakata M, Koshiba H, Eto K, Nance WE (1980) A genetic study of anodontia in X-linked hypohidrotic ectodermal dysplasia. Am J Hum Genet 32: 908–919. 7446529 PMC1686146

[33] Lexner MO, Bardow A, Juncker I, Jensen LG, Almer L, et al (2008) X-linked hypohidrotic ectodermal dysplasia. Genetic and dental findings in 67 Danish patients from 19 families. Clin Genet 74: 252–259. 18510547 10.1111/j.1399-0004.2008.01037.x

[34] Wang SK, Chan HC, Makovey I, Simmer JP, Hu JC (2012) Novel PAX9 and COL1A2 missense mutations causing tooth agenesis and OI/DGI without skeletal abnormalities. PLoS One 7: e51533. 23227268 10.1371/journal.pone.0051533 PMC3515487

[35] Bhuiyan ZA, Jongbloed JD, van der Smagt J, Lombardi PM, Wiesfeld AC, et al (2009) Desmoglein-2 and desmocollin-2 mutations in dutch arrhythmogenic right ventricular dysplasia/cardiomypathy patients: results from a multicenter study. Circ Cardiovasc Genet 2: 418–427. 20031616 10.1161/CIRCGENETICS.108.839829

[36] Morak M, Massdorf T, Sykora H, Kerscher M, Holinski-Feder E (2011) First evidence for digenic inheritance in hereditary colorectal cancer by mutations in the base excision repair genes. Eur J Cancer 47: 1046–1055. 21195604 10.1016/j.ejca.2010.11.016

[37] El Hokayem J, Huber C, Couvé A, Aziza J, Baujat G, et al (2012) NEK1 and DYNC2H1 are both involved in short rib polydactyly Majewski type but not in Beemer Langer cases. Journal of Medical Genetics 49: 227–233. 22499340 10.1136/jmedgenet-2011-100717

[38] Liu F, Chu EY, Watt B, Zhang Y, Gallant NM, et al (2008) Wnt/beta-catenin signaling directs multiple stages of tooth morphogenesis. Dev Biol 313: 210–224. 18022614 10.1016/j.ydbio.2007.10.016 PMC2843623

[39] Fliniaux I, Mikkola ML, Lefebvre S, Thesleff I (2008) Identification of dkk4 as a target of Eda-A1/Edar pathway reveals an unexpected role of ectodysplasin as inhibitor of Wnt signalling in ectodermal placodes. Dev Biol 320: 60–71. 18508042 10.1016/j.ydbio.2008.04.023

[40] Laurikkala J, Mikkola M, Mustonen T, Aberg T, Koppinen P, et al (2001) TNF signaling via the ligand-receptor pair ectodysplasin and edar controls the function of epithelial signaling centers and is regulated by Wnt and activin during tooth organogenesis. Dev Biol 229: 443–455. 11203701 10.1006/dbio.2000.9955

