# Supplementary material for: Involvement of and Interaction between WNT10A and EDA Mutations in Tooth Agenesis Cases in the Chinese Population
Source: PLoS One. 2013 Nov 27;8(11):e80393. doi: 10.1371/journal.pone.0080393 (PMC3842385; doi:10.1371/journal.pone.0080393)
Supplement: Table S1 — Single nucleotide polymorphisms in EDAR and EDARADD of 114 patients. (PPT) [file pone.0080393.s001.ppt]

## Slide 1
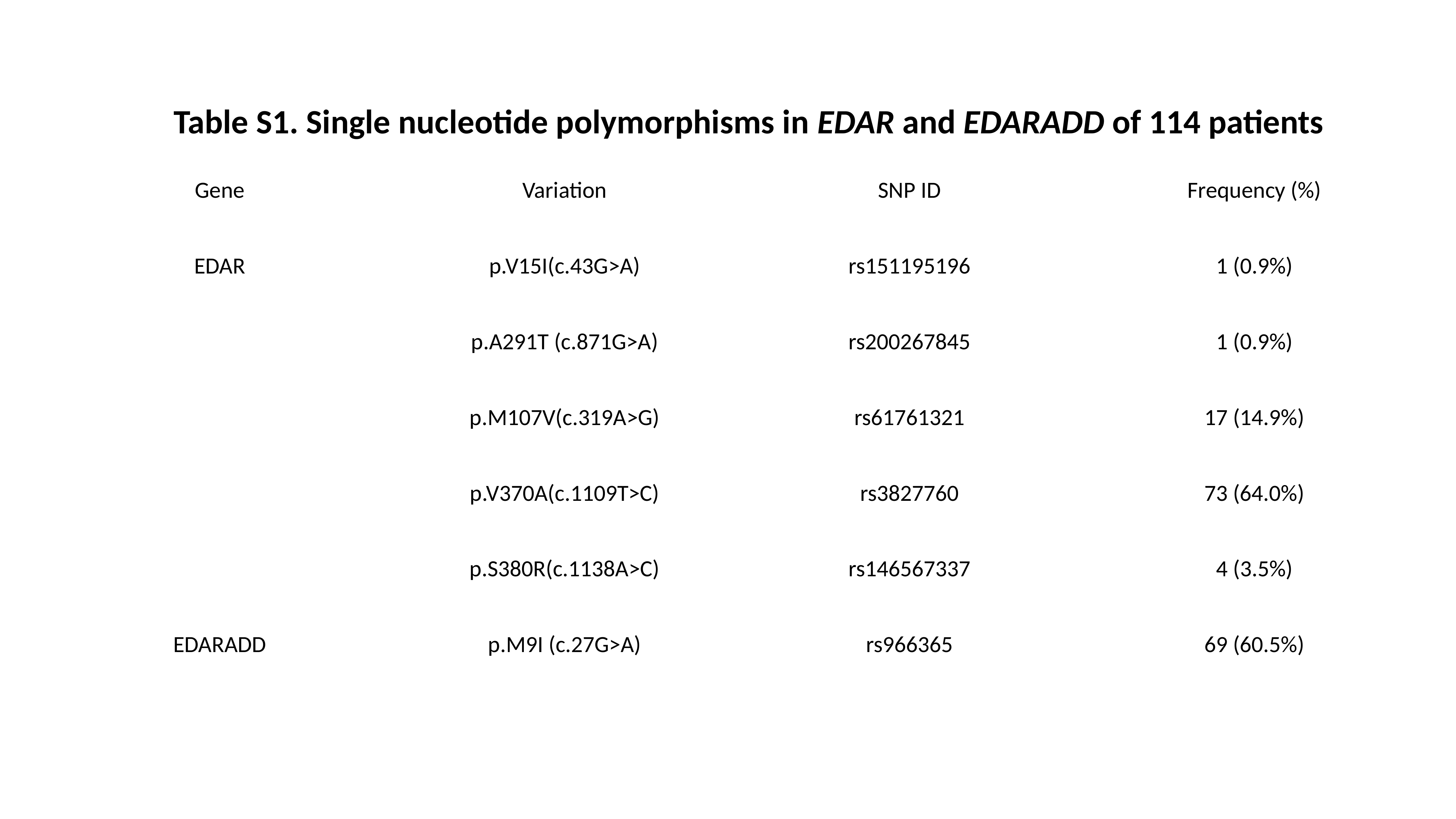

Table S1. Single nucleotide polymorphisms in EDAR and EDARADD of 114 patients
| Gene | Variation | SNP ID | Frequency (%) |
| --- | --- | --- | --- |
| EDAR | p.V15I(c.43G>A) | rs151195196 | 1 (0.9%) |
| | p.A291T (c.871G>A) | rs200267845 | 1 (0.9%) |
| | p.M107V(c.319A>G) | rs61761321 | 17 (14.9%) |
| | p.V370A(c.1109T>C) | rs3827760 | 73 (64.0%) |
| | p.S380R(c.1138A>C) | rs146567337 | 4 (3.5%) |
| EDARADD | p.M9I (c.27G>A) | rs966365 | 69 (60.5%) |
